# Highly Enhancing the Interfacial and Mechanical Properties of Basalt Fiber/Poly(phthalazinone ether nitrile ketone) Composite by Thermoplastic Sizing Agents with Different Structures

**DOI:** 10.3390/polym14142947

**Published:** 2022-07-21

**Authors:** Hang Jia, Cheng Liu, Yue Qiao, Yu Zhang, Kaiyuan Fan, Manxia Zhang, Xigao Jian

**Affiliations:** 1 State Key Laboratory of Fine Chemicals, Dalian University of Technology, Dalian 116024, China; POLYJH@mail.dlut.edu.cn (H.J.); qiao123@mail.dlut.edu.cn (Y.Q.); zhangyu25@mail.dlut.edu.cn (Y.Z.); 18266003166@mail.dlut.edu.cn (K.F.); jian4616@dlut.edu.cn (X.J.); 2 Department of Polymer Science & Materials, Dalian University of Technology, Dalian 116024, China; 3 Dalian Basalt Fiber Resin Matrix Composite Engineering Research Center, Dalian 116012, China; 4 Department of Environmental Science & Engineering, Dalian Maritime University, Dalian 116026, China; zhangmanxia@163.com

**Keywords:** basalt fiber, high-performance thermoplastic resin, composite, interfacial modification

## Abstract

The interfacial modification of basalt-fiber-reinforced polymer (BFRP) composites is an essential research field and many techniques have been developed to improve the adhesion between basalt fiber (BF) and the matrix. However, most studies were based on the matrixes of general plastics and epoxy resins. In this work, five different chain structures of thermoplastic sizing agents were used to improve the interfacial properties of unidirectional BF-reinforced soluble and high-temperature-resistant poly(phthalazinone ether nitrile ketone) (BF/PPENK) composites. DMA results showed that the poly(ether nitrile) (PEN)-sized BF/PPENK (BF-PEN/PPENK) composite exhibited the optimal interfacial performance, with a storage modulus (*E*′) and glass transition temperature (*T_g_*) up to 50 GPa and 288 °C, respectively. Moreover, the tensile strength, compressive strength, flexural strength, and interlaminar shear strength of the BF-PEN/PPENK composite reached 778 MPa, 600 MPa, 1115 MPa and 57 MPa, respectively, and increased by 42%, 49%, 20% and 30% compared with the desized BF/PPENK composite. This study provides some suggestions for the design of sizing agents to modify the interface of BF and high-performance thermoplastic resin.

## 1. Introduction

Fiber-reinforced polymer (FRP) composites, such as carbon fiber [1], glass fiber [2], aramid fiber [3], ultra-high-molecular-weight polyethylene fiber [4], polypropylene fiber [5] and basalt fiber [6], etc., have obtained wide range of applications for their light weight, high strength, impact resistance, corrosion resistance. In particular, basalt fiber (BF) possesses some favorable properties, such as non-pollution, low cost, high- and low-temperature resistance, chemical stability, thermal insulation, sound absorption, and flame-retardant nature, and especially excellent mechanical properties [7,8,9,10,11]. Basalt-fiber-reinforced polymer (BFRP) composites, combining the advantages of the fiber and resin, have great potential in the fields of building, aerospace, petrochemical, automobile, marine, energy conservation, and environmental protection [12,13,14,15]. However, the surface of BF is quite smooth and inert, and the interfacial adhesion between the fiber and resin is weak [16,17,18]. Therefore, various strategies have been used to construct a reasonable interfacial structure of the BFRP composites to improve their interfacial performance.

The sizing treatment of the fiber surface is a simple, easy-to-operate and industrialized strategy, which mainly includes silane coupling-agent modification, polymer sizing, nanoparticle adhesion, etc. [19,20,21]. Among them, polymer sizing has been regarded as a standardized and commercially available method of fiber surface treatment during the manufacturing process of commercial fibers [22], due to its advantages of preventing the fluffs and yarn breakage without damaging the surface and properties of fibers [23,24,25]. However, most commercial polymer sizing agents are variously diluted epoxy resins, whose degradation temperatures (about 250 °C) are much lower than the processing temperatures of high-performance engineering plastics [26,27,28]. This would inevitably result in the degradation of the polymer coating on the fiber surface under the high temperature of composite processing and, thus, the defects on the interface of the fiber and heat-resistant thermoplastic resin [29]. Furthermore, the compatibility between the epoxy thermoset (TS) resin and heat-resistant thermoplastic (TP) matrix is not very high, which has a passive effect on the interfacial properties of composites. Therefore, the design and synthesis of heat-resistant thermoplastic sizing agents are particularly important for the development and application of high-performance thermoplastic polymer composites.

The polymer sizing agents generally used are polymers with relatively low molecular weight (LMW), and their modulus is lower than that of the fiber and matrix. According to the “deformation layer theory” [30], if the polymer sizing agent has good compatibility with the resin matrix, it will form an interfacial deformation layer to better buffer the direct stress impact on the fibers. Therefore, considering the high-temperature processing of fiber-reinforced high-performance thermoplastic polymer (FRHTP) composites and the extreme service environment, polymer sizing agents should have the following characteristics: high-temperature resistance, certain compatibility with the matrix, good solubility, or easy dispersibility.

At present, many efforts have been focused on modifying the interface of fiber-reinforced poly(ether ether ketone) (PEEK) composites by designing oligomeric high-performance polymers as sizing agents, which have similar chemical structures and good compatibility to the PEEK matrix. Zhu et al. [31] established an effective interfacial enhancement method for GF/PEEK composites by introducing aminated poly(ether ether ketone) on the surface of the glass fibers. Wang et al. [32] and Hassan et al. [33] reported a transformed fiber surface morphology and chemical composition with heterocyclic poly(aryl indole ketone) and soluble poly(ether ketone ketone) separately, to improve the interfacial properties of CF/PEEK composites. Wang et al. [34] used polyimide (PI) to modify the surface of short carbon fibers (SCF), and the tensile strength and bending strength of PI-coated SCF/PEEK composites showed improvements of 11.8% and 16.6%, respectively, compared with commercial cases. However, these modification methods all require the pre-activation of the fibers, which inevitably causes the fibers to be damaged, and the synthesis and treating processes are also complicated.

In addition, until now, there are still very few reports on the interfacial properties of basalt-fiber-reinforced high-performance thermoplastic polymer (BFRHTP) composites, which hinders the application and development of basalt fiber in high-end manufacturing industries. This may be because of the smoother and more inert surface of BF compared with CF, and the difficulty of balancing its surface activation and fiber strength. Therefore, it is significant to explore the influence of structures of polymer sizing agents on the interfacial properties of BFRHTP composites.

In this work, five low-molecular-weight (LMW) thermoplastic polymers (${\bar{M}}_{n}$ = 10,000 g/mol), namely polyvinylpyrrolidone (PVP), polyether nitrile (PEN), poly(phthalazinone ether ketone) (PPEK), poly(phthalazinone ether nitrile) (PPEN), and poly(phthalazinone ether nitrile ketone) (PPENK), were used as sizing agents for the surface treatment of basalt fibers. The chains of PEN, PPEK, PPENK, and PPEN have aromatic rigid structures, which are similar to the matrix, but their cyano group content and flexibility of them are different. Additionally, PVP with an aliphatic structure has better flexibility than the other four sizing agents. Then, the effect of the chain structures of the sizing agent on the interfacial and mechanical properties of BF/PPENK composites was investigated in detail. Furthermore, the solubility parameters of the sizing agents and the PPENK matrix resin were calculated by the group contribution method, and their compatibilities were analyzed by these parameters. XPS and SEM were used to characterize the chemical composition and morphology of the BF surfaces, respectively. The dynamic thermomechanical analysis (DMA) was performed to reveal the thermal property and interfacial adhesion of the BF/PPENK composites. A tensile test, compression test, bending test, and interlayer shear test were employed to evaluate the mechanical properties of the BF/PPENK composites. The failure modes and mechanisms of the tensile and compression test were analyzed by the optical microscope and SEM. This study provides some suggestions for the design of sizing agents to modify the interface of BF and high-performance thermoplastic resin.

## 2. Materials and Experiments

### 2.1. Materials

Continuous basalt fiber (untwisted roving; diameter, 9 μm; density, 2.8 g/cm^3^; tensile strength and modulus, 2.3 GPa and 84 GPa, respectively) was kindly provided by Guizhou Shixin Basalt Technology Co., Ltd., Guiyang, China. Poly(phthalazinone ether nitrile ketone) (PPENK, nitrile: ketone, 1:1; intrinsic viscosity, 0.44 dL/g) resin and 4-(4-hydroxyphenyl)phthalazinone (DHPZ) were purchased from Dalian Polymer New Material Co., Ltd., Dalian, China. 1,3-Dihydroxybenzene (DHB), 2,6-difluorobenzonitrile (DFBN), 4,4′-difluorophenyl ketone (DFK) and polyvinylpyrrolidone (PVP, K13–18, =10,000 g/mol) were obtained by Shanghai Aladdin Bio-Chem Technology Co., Ltd., China. Anhydrous potassium carbonate (K_2_CO_3_), N,N-dimethylacetamide (DMAc), N-methylpyrrolidone (NMP), toluene, ethanol, and acetone were obtained from Sinopharm Chemical Reagent Co., Ltd., Shanghai, China and used directly without any treatment.

### 2.2. Synthesis of the TP Sizing Agents

**Scheme 1:**
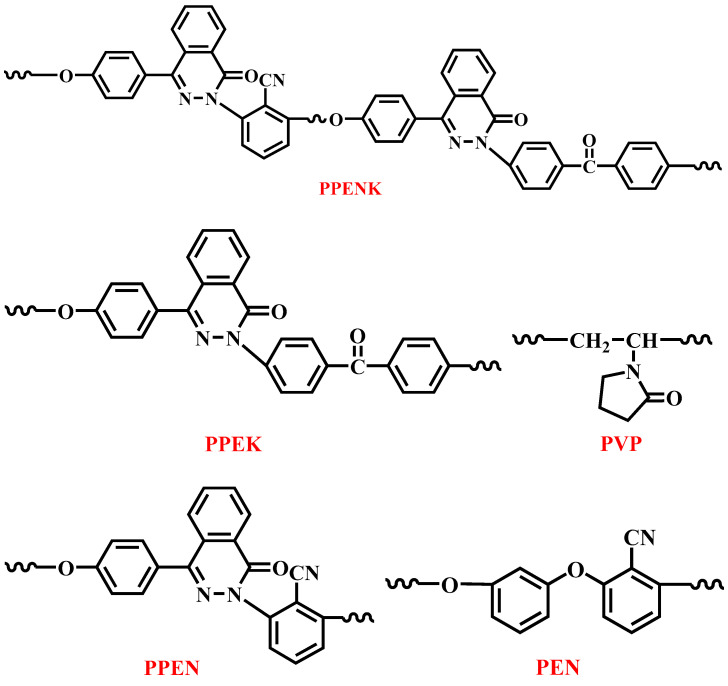
Chemical structures of PPENK, PPEK, PVP, PPEN and PEN.

The chemical structures of PPENK, PPEK, PVP, PPEN and PEN are exhibited in Scheme 1, and their 5% thermal-decomposition temperatures are shown in Figure S1, which all meet the processing requirement. The number-average molecular weight of the designed polymers was 10,000 g/mol, with a theoretical DP of 49, 53, 63, 95 and 103 for PPEK, PPENK, PPEN, PEN and PVP, respectively. Except for PVP, the other four LMW sizing agents, PPEK, PPEN, PPENK (N:K = 1:1) and PEN, were synthesized according to the similar procedure in our previous works [35,36]. Taking the LMW PEN, for instance, a typical principle of polymerization was performed as shown in Figure S2. DHB (0.09792 mol, 10.9667 g), DFBN (0.1 mol, 13.9110 g), anhydrous K_2_CO_3_ (0.14 mol, 19.3494 g), 25 mL NMP and 45 mL toluene were added to a 250 mL three-necked round-bottom flask outfitted with a mechanical stirrer, a nitrogen inlet, a Dean–Stark trap outfitted with a condenser, and a nitrogen inlet and outlet. Firstly, the mixture was stirred at 130 °C for 2 h under an N_2_ atmosphere to distill the resultant water azeotropically, and then the toluene was distilled off. Subsequently, the mixture was stirred at 160 °C for 8 h. The resultant viscous mixture was poured into hot deionized water with vigorous stirring, and the precipitated product was rinsed with hot deionized water three times, and then dried under a vacuum at 120 °C for 24 h.

### 2.3. Preparation of Composites

Acetone was used to remove the pristine sizing and pollutants on the surface of BFs for 48 h [37]. Then, they were cleaned with deionized water and dried in a vacuum oven at 120 °C for 4 h to obtain the desized BF. The LMW PVP, PEN, PPEK, PPEN and PPENK were separately dispersed in NMP, and the ratio of solid to solvent was 1 g/100 mL. Then, after ultrasonication (5 min), standing (15 min) and drying (150 °C, 30 min), the desized BFs were covered with LMW polymer coating, named BF-PVP, BF-PEN, BF-PPEK, BF-PPEN and BF-PPENK, respectively. The resized BFs were placed in a dipping tank (the ratio of solute mass to solvent volume is 0.16 g/mL), and the BF/PPENK prepregs were prepared by the solution dipping method. Finally, the dried prepregs were cut into a suitable size, and the unidirectional composite laminates were prepared through a vacuum thermocompression up to 320 °C with a pressure of 7 MPa (as is shown in Figure S3), named BF-PVP/PPENK, BF-PEN/PPENK, BF-PPEK/PPENK, BF-PPEN/PPENK and BF-PPENK/PPENK, respectively. The volume fraction of BFs in the BF/PPENK composites was about 43% (mass fraction was about 60%).

### 2.4. Characterization

X-ray photoelectron spectroscopy (XPS, ESCALAB250Xi; optimum sensitivity: 1,000,000 cps (Mono XPS, Ag 3d5/2)) was used to characterize the surface chemical element compositions of different surfaces of the modified BF. Scanning electron microscopy (SEM, SU8200; loading voltage and current: 5 kV and 10 µmA; SEI resolution ratio: 0.8 nm) was employed to investigate the microscopic surface of BFs and the fracture morphologies of BF/PPENK composites after the tensile test and compression test. The optical microscope (Nikon, ECLIPSE LV100ND) was used to observe the side of the polished compressive failing sample and study its failing mode (the magnification was 50 times).

The thermal stability of the sizing agents was measured by thermal gravimetric analysis (TGA, METTLER TOLE-DO TGA1) at a heating rate of 20 °C/min from 30 °C to 800 °C (after being kept at 150 °C for 10 min) under a nitrogen atmosphere. Dynamic mechanical analysis (DMA, sample size: 40 × 6 × 2 mm^3^) measurement was carried out on METTLER TOLEDO DMA/SDTA861e at a single cantilever clamp, 1 Hz, 3 N, and a heating rate of 3 °C/min, from 30 °C to 350 °C under air atmosphere. 

Tensile test (sample size: 250 × 15 × 1.5 mm^3^), compression test (sample size: 80 × 12.5 × 2 mm^3^), flexural test (sample size: 80 × 12.7 × 2 mm^3^), and interlaminar shear strength test (sample size: 20 × 10 × 2 mm^3^) of the BF/PPENK composites were determined on a universal testing machine (Instron 5982; 100 kN force sensor) according to ASTM D3039, SACMA SRM 1R, ASTM D790 and ISO 14130, respectively. At least five parallel measurements were conducted and averaged for each final result.

## 3. Results and Discussion

### 3.1. Compatibility of Polymer Sizing Agents and PPENK Matrix

The group contribution method was used to calculate the solubility parameters of LMW PVP, PEN, PPEK, PPEN and PPENK. The results of solubility parameters and their different components are shown in Table 1. According to the principle of similar solubility parameters, we compared the differences in total solubility parameters (|*δ_t_* − *δ_t,matrix_*|) between each polymer sizing agent and matrix resin (*δ_t,matrix_*) to preliminarily determine their respective compatibility. The calculation indicated that the |*δ_t_* − *δ_t,matrix_*| of PVP, PEN, PPEK, PPEN and PPENK are 0.58, 0.28, 0.26, 0.35 and 0, respectively. These differences are very small, indicating the good compatibility between these sizing agents and the PPENK matrix, and further sorting the compatible degree of them obtained the order PPENK > PPEK > PEN > PPEN > PVP. However, the differences in polarization components (|*δ_p_* − *δ_p,matrix_*|) between the PVP, PEN, PPEK, PPEN and PPENK and the matrix are 5.35, 1.34, 0.8, 0.95 and 0, respectively. According to the “similar polarity principle” and “structural similarity principle”, the compatibility of PEN with the matrix is weaker than PPEN. Therefore, the final compatible degree of them is PPENK > PPEK > PPEN > PEN > PVP.

### 3.2. The Surface Morphology and Elements of BFs

**Figure 1:**
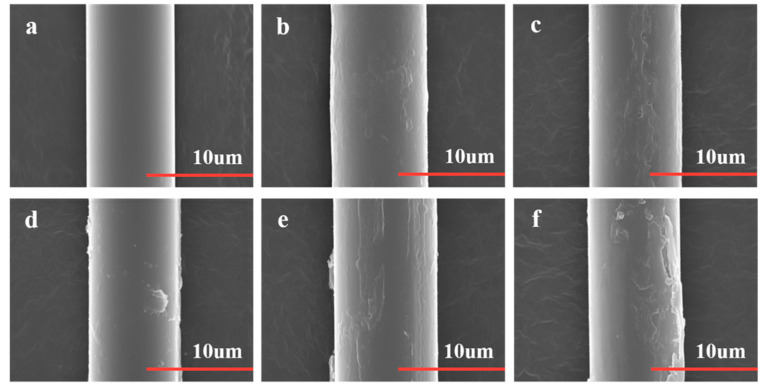
SEM images of (**a**) BF—Desized, (**b**) BF-PVP, (**c**) BF-PEN, (**d**) BF-PPEK, (**e**) BF-PPEN and (**f**) BF-PPENK.

SEM images for the BFs treated with different sizing agents are shown in Figure 1. The surface of the desized BF treated with acetone is considerably smooth (Figure 1a), and it is chemically inert and cannot form chemical bonds with the polymer matrix. Therefore, the BFs without any surface treatment have difficulty in forming a dependable interfacial phase with PPENK. As illustrated in Figure 1b–f, the surface morphologies of the resized BFs are much rougher than the BF—desized. Furthermore, their roughness and morphology are different, which can be attributed to the film-forming capacity of these TP sizing agents on the BF surface. According to the morphologies of the resized BF surfaces, the uniformity, smoothness and integrity were used as the criteria for judging the film-forming ability, in the order of PVP > PEN > PPEN > PPEK > PPENK. This may be ascribed to the different flexibility of molecular chains and their adhesion with BF.

The high-resolution XPS *N*(*1s*) peaks for the desized and resized BFs are shown in Figure S4b. It should be noted that the desized BF had only a weak N-H bonding at 399.7 eV [38]. However, the *N1s* peaks of BF-PVP were deconvoluted into two specific peaks at 399.4 eV (pyrrolic N) and 400.1 eV (O=C-N). Additionally, the signal of the C≡N bond at 399.35–399.55 eV appears for BF-PEN, BF-PPEN and BF-PPENK. Furthermore, the characteristic peaks at 399 eV, 399.25–399.95 eV, 399.95–400.8 eV and 400.9–401.9 eV are associated with C-N, N-N, C=N and O=C-N bonds in the phthalazinone structure, respectively. The chemical compositions of *C*(*1s*), *O*(*1s*), and *N*(*1s*) were examined with reference to *Al*(*2p*), which is absent in the TP sizing agents. The results indicated that the resized BFs had a higher ratio of O/Al and N/Al compared with the desized BF (Table S2). In particular, the BF-PEN surfaces have the highest contents of N and O elements and polarity, deriving from cyano and ether bonds, because of the high-number-average DP of PEN. Similarly, the high content of N and O elements on the surface of BF-PVP comes from the abundant pyrrolidone rings, also giving it higher polarity. However, the content of N and O elements on the surfaces of BF-PPEK, BF-PPEN and BF-PPENK decreased greatly, which was more related to their low-number-average DP. Therefore, combined with the SEM results, five sizing agents were successfully coated on the BF surface and showed different surface morphologies and elemental characteristics.

### 3.3. Interfacial Properties

**Figure 2:**
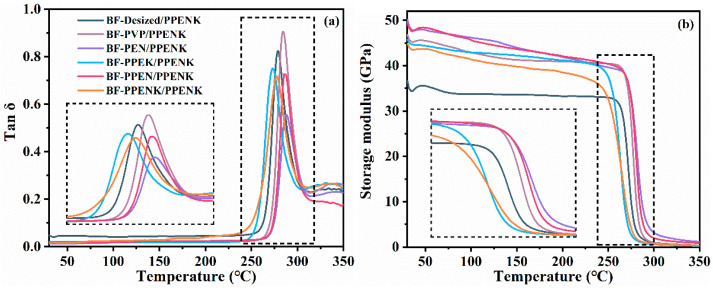
Dynamic thermomechanical analysis of the BF—Desized/PPENK, BF-PVP/PPENK, BF-PEN/PPENK, BF-PPEK/PPENK, BF-PPEN/PPENK and BF-PPENK/PPENK composites; (**a**) loss factor curves and (**b**) storage modulus curves.

The loss factor (tan*δ*) is defined as the ratio of the loss modulus to the storage modulus, and represents the ability of materials to lose energy by alternate load [39] and the peak temperature of the tan*δ* curve is the glass transition temperature (*T_g_*). Moreover, the tan*δ* peak value (tan*δ_max_*) can also be used to evaluate the interfacial adhesion of the resin matrix composites; namely, the lower the tan*δ_max_* value, the better interfacial adhesion [39,40]. As shown in Figure 2a, except for BF-PVP/PPENK, the tan*δ_max_* values of the other four sizing-modified BF/PPENK composites were lower than the BF—Desized/PPENK composite, indicating the improved interfacial properties between the resized BFs and the PPENK matrix. Furthermore, the *T_g_* of the BF-PVP/PPENK was increased by 6 °C compared with that of the BF—Desized/PPENK composite, due to the enhanced interfacial properties. Additionally, the increased tan*δ_max_* for BF-PVP/PPENK may be ascribed to the much lower *T_g_* of interfacial PVP than the PPENK matrix and its higher viscosity at its elastomeric state (285 °C) than other sizing agents with higher *T_g_*, as evidenced by its largest loss modulus and loss factor (Figure S5a). Moreover, the improved mobility of the interfacial layer caused the declined *T_g_* of BF-PPEK/PPENK and BF-PPENK/PPENK composites, which may be related to the low-number-average DP and weak polarity of the sizing agents. Therefore, the lowest tan*δ_max_* (0.5559) and the highest *T_g_* (288 °C) demonstrated that PEN was the most effective sizing agent for improving the interfacial phase structure of the BF/PPENK composites than the others (Table S3). 

Moreover, Zorowski and Murayama [41] proposed that the tan*δ* of the composite can be divided into two parts of the energy dissipation at the individual components and the interface. The loss factor of a composite can be stated as:

(1) $$\tan\delta_{c}=\tan\delta_{s}+\tan\delta_{in}$$

where tan*δ_c_*, tan*δ_s_* and tan*δ_in_* are loss factors of the composite, the single component (PPENK and BF) and the interface, respectively.

However, tan*δ_in_* cannot be measured directly, and can only be calculated by taking the difference between tan*δ_c_* and tan*δ_s_*, so as to further evaluate the interfacial properties of the composite. The tan*δ_s_* can be obtained from the complex modulus of the fiber and matrix according to the following formula:

(2) $$\tan\delta_{s}=\frac{\tan\delta_{f}\cdot E_{f}^{\prime }\cdot V_{f}}{E_{c}^{\prime }}+\frac{\tan\delta_{m}\cdot E_{m}^{\prime }\cdot V_{m}}{E_{c}^{\prime }}$$

where the tan*δ_m_*, *E*′*_m_* and *V_m_* are the loss factor, storage modulus and volume fraction of PPENK, and the tan*δ_f_*, *E*′*_f_*, and *V_f_* are the loss factor, storage modulus and volume fraction of the BF, respectively. 

Compared with the tan*δ_m_*, the tan*δ_f_* would be almost zero, because of the much higher stiffness of BF than the matrix. Therefore, the tan*δ_s_* is only determined by the tan*δ_m_*, *E*′*_m_*, *V_m_* and *E*′*_c_*, and the tan*δ_in_* can be represented by the following formula:

(3) $$\tan\delta_{in}=\tan\delta_{c}-\frac{\tan\delta_{m}\cdot E_{m}^{\prime }\cdot V_{m}}{E_{c}^{\prime }}$$

**Scheme 2:**
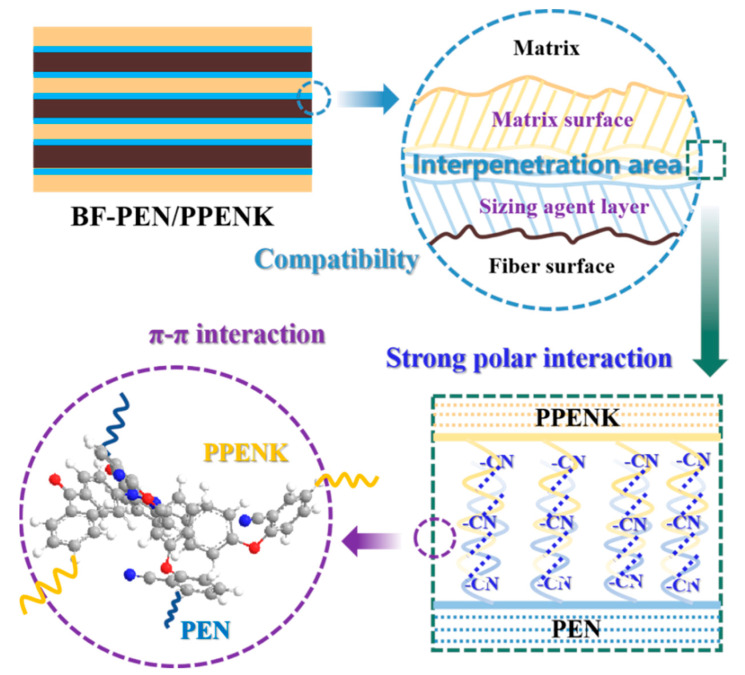
Schematic diagram of the PEN sizing agent enhancing the BF/PPENK composite interfacial properties.

The *E*′*_m_* and tan*δ_m_* were measured in our previous work [42], and the tan*δ_in_* curves are shown in Figure S5b. The lower interfacial loss represents the better interfacial bonding ability of the composite [43]. Therefore, the BF-PEN/PPENK composite may have the highest interfacial bonding strength among these resized BF/PPENK composites. The great improvement in interfacial properties may be attributed to the strong polar interaction, *π*-*π* interaction, and good compatibility of the LMW PEN agent and PPENK matrix (Scheme 2).

Storage modulus (*E*′) is the contribution of the elastic component of composites, and is primarily determined by the matrix, fiber and interfacial adhesion. As the temperature increased, the *E*′ of the BF/PPENK composites decreased slowly before *T_g_* (Figure 2b), because of the relaxation process in the polymer matrix. The initial *E*′ of the BF-PVP/PPENK, BF-PEN/PPENK and BF-PPENK/PPENK composites was 46.78 GPa, 50.14 GPa and 49.50 GPa, which were increased by 27.6%, 36.8% and 35.1% compared with BF—Desized/PPENK (36.65 GPa), respectively, and their *E*′ values were higher than the BF—Desized/PPENK in the whole test temperature range. Additionally, the initial *E*′ of the BF-PPEK/PPENK and BF-PPENK/PPENK composites also reached 45.74 GPa and 44.81 GPa, respectively. However, their *E*′ curves were located below the BF—Desized/PPENK after 250 °C, which is consistent with the results obtained from the tan*δ* curves. In conclusion, constructing a flexible interfacial phase structure for the BF/PPENK composites is a very effective interfacial modification strategy. In addition, combined with the compatibility results, it can be found that the polar interaction plays a more important role than compatibility between the sizing agent and matrix for improving the interfacial properties of BF/PPENK composites.

### 3.4. Tensile Properties

**Figure 3:**
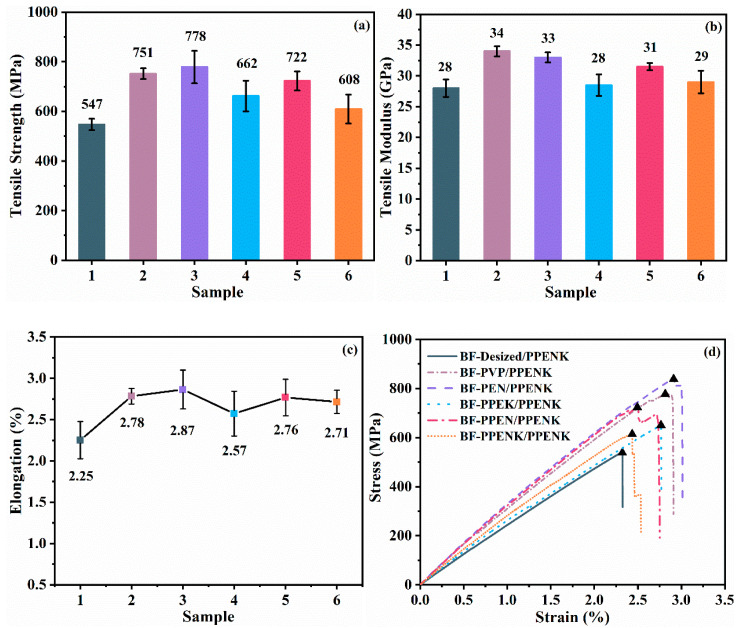
Tensile properties of the 1: BF—Desized/PPENK, 2: BF-PVP/PPENK, 3: BF-PEN/PPENK, 4: BF-PPEK/PPENK, 5: BF-PPEN/PPENK and 6: BF-PPENK/PPENK composites; (**a**) strength, (**b**) modulus, (**c**) breaking strain and (**d**) stress–strain curves.

As illustrated in Figure 3, all the values of tensile strength, modulus and elongation for the resized BF/PPENK composites are much higher than those of the desized BF/PPENK composite (547 MPa, 28 GPa and 2.25%), which may be attributed to the enhanced interfacial adhesion. The BF-PEN/PPENK composite, which has the highest interfacial properties, displayed the tensile strength, modulus and elongation at break of 778 MPa, 33 GPa and 2.87%, about 42%, 18% and 28% higher than those of desized BF/PPENK, respectively. In addition, it is worth mentioning that BF-PVP/PPENK exhibited a high tensile strength, modulus and elongation at break of 751 MPa, 34 GPa and 2.78%, about 37%, 21% and 24%, respectively, which further proved that the flexible interfacial phase can effectively retard the crack propagation and improve the interfacial properties of the composites. Furthermore, as for the other three composites with similar sizing agents containing a phthalazinone structure, BF-PPEN/PPENK exhibited higher tensile strength, tensile modulus and elongation at break of 722 MPa, 31 GPa and 2.76%, respectively, than those of BF-PPEK/PPENK and BF-PPENK/PPENK. This may be due to the relatively better film-forming properties and more polar cyano groups (−CN) of PPEN than those of PPEK and PPENK. 

### 3.5. Tensile Failure Mechanism

**Figure 4:**
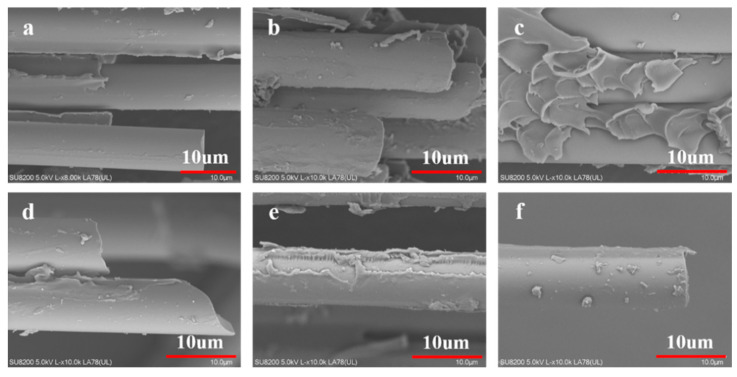
SEM images of tensile test fracture; (**a**) BF—Desized/PPENK, (**b**) BF-PVP/PPENK, (**c**) BF-PEN/PPENK, (**d**) BF-PPEK/PPENK, (**e**) BF-PPEN/PPENK and (**f**) BF-PPENK/PPENK composites.

In the longitudinal tensile test of unidirectional composites, the damage expansion can be divided into four types: (1) interface debonding of the fiber and matrix; (2) the cracks in the fiber extend to the matrix; (3) the matrix deformation and ductile fracture; (4) sequential fracture of the adjacent fibers. Additionally, different damage expansion forms and processes will lead to three kinds of typical failure modes: (1) the bundle failing model; (2) the fracture failing model; (3) the accumulated damage model. Figure S6 displays the macro-morphologies of tensile failure modes for the resultant composites. On the whole, the tensile failure modes of all the BF/PPENK composites are the bundle failing model, which can be attributed to the poor tow-spreading condition of the BF bundles. However, their failure morphologies were different, and the number of cracks on the specimens had a certain positive correlation with the tensile strength. The BF—Desized/PPENK composite has the least cracks, which is related to its poor interfacial strength and the inferior wettability of the PPENK matrix on BF. In contrast, the resized BF/PPENK composites split into more fragments, due to their improved wettability of BF and interfacial properties. The SEM images reflected the much more microscopic failure mechanisms. As shown in Figure 4a, the surface of the pull-out fiber was smooth, which confirmed that the main failure mechanism of BF—Desized/PPENK was interface-debonding failure. For the BF-PVP/PPENK, BF-PPEK/PPENK and BF-PPENK/PPENK composites, the surface roughness and the resin content of the pull-out fiber surface were increased, but no obvious shear damage morphology appeared for the matrix (Figure 4b,d,f), indicating that the cracks propagated along with the fiber in the interfacial phase. In this case, the tightly adhesive matrix on the BF surface can effectively transfer the load, resulting in improved tensile strength to a certain extent. As for the BF-PVP/PPENK composite, the significantly increased tensile strength may be related to the strong interfacial flexibility and polarity. In particular, BF-PEN/PPENK has the obvious “fish-scale” morphology (Figure 4c), and the main tensile-failure mechanisms were matrix deformation and ductile fracture, because of the strong interfacial adhesion. The mechanism of the BF-PPEN/PPENK composite is similar to that of BF-PEN/PPENK, but the matrix resin deformation is not obvious because of the relatively low number-average DP, weak polarity and rigid chain structures of PPEN.

### 3.6. Compressive Properties

**Figure 5:**
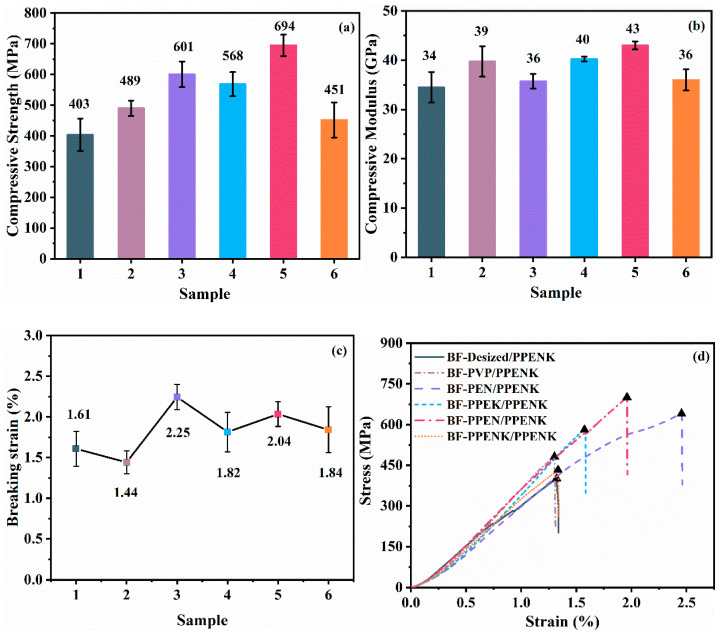
Compressive properties of the 1: BF—Desized/PPENK, 2: BF-PVP/PPENK, 3: BF-PEN/PPENK, 4: BF-PPEK/PPENK, 5: BF-PPEN/PPENK and 6: BF-PPENK/PPENK composites; (**a**) strength, (**b**) modulus, (**c**) breaking strain and (**d**) stress–strain curves.

The compressive properties of the BF/PPENK composites are shown in Figure 5. The compressive strength and modulus of the BF-PVP/PPENK composite were slightly increased to 489 MPa and 39 GPa compared with BF—Desized/PPENK (403 MPa and 34 GPa). This increase was less obvious than that of the tensile strength, probably due to the much smaller size and larger load of samples for the compressive test than those in the tensile test. That is, when the compression load reaches a very large level (more than 20 kN), the flexible interfacial phase is destroyed in a moment, and the ability of the flexible layer to retard crack propagation is greatly weakened, so the breaking strain of the BF-PVP/PPENK composite was decreased. Compared with the BF—Desized/PPENK, the compressive strengths of BF-PEN/PPENK and BF-PPEN/PPENK composites are 601 MPa and 694 MPa, increased by 49% and 72%, respectively. On the one hand, this may be related to the strong polar interaction of the cyano groups, resulting in the enhanced interfacial strength. On the other hand, the molecular chains of aromatic structures have strong rigidity and can withstand higher loads. Due to the stronger rigidity of the PPEN chain than the PEN chain, the modulus of BF-PPEN/PPENK (43 GPa) is higher than BF-PEN/PPENK (36 GPa), while the breaking strain of BF-PPEN/PPENK (2.04%) is lower than that of BF-PEN/PPENK (2.25%). This is also reflected in the compressive properties of the BF-PPEK/PPENK composite, whose strength, modulus and breaking strain are improved to 568 MPa, 40 GPa and 1.82%, respectively. The mildly enhanced compressive properties of BF-PPENK/PPENK composites mainly come from the improvement in wettability of the resized BF with PPENK. Owing to the rigid structure and low number-average DP of the PPENK sizing agent, its film-forming property is too poor to form a reasonable interfacial phase structure. On the whole, the appropriate content of the rigid structure in the sizing agent is necessary to endow the interfacial phase with a certain strength, which means that the good film-forming ability and proper flexibility of the sizing agent are significant for the interfacial phase of BF-reinforced high-performance thermoplastic composites.

**Figure 6:**
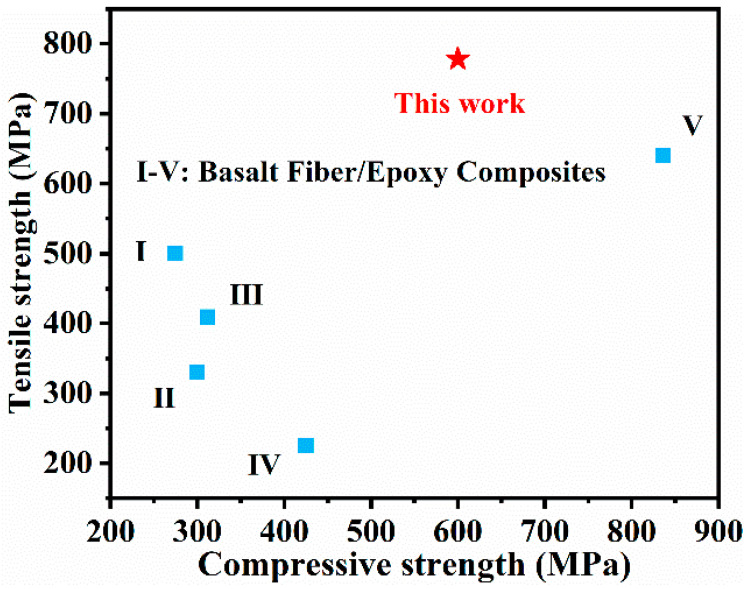
Comparison of tensile and compressive strength of BF-PEN/PPENK with BF/EP composites (I–V: [44,45,46,47,48]).

In order to clarify the position of the BF/PPENK composites in the field of BFRP, and to highlight the advancement of this work, the tensile and compressive strengths were compared with those reported in references [44,45,46,47,48]. As shown in Figure 6, this work achieved good results compared with basalt-fiber-fabric-reinforced epoxy resin (BF/EP) composites.

### 3.7. Compressive Failure Mechanism

The compressive failure modes of FRP composites are relatively complex, and the common forms include delamination, micro-buckling, shear fracture, kink band and bundle splitting [49,50]. Generally, the final compressive failure model of each composite laminate is a combination of them. Among the many influencing factors, fiber, matrix and interfacial phase structure are the most complex and critical [51]. Madhukar et al. [52] found that the compressive failure mechanism depended strongly on the interface conditions; that is, as the interfacial strength increased, and the compression failure mode changed from delamination and buckling to bundle splitting to fiber compressive failure.

**Figure 7:**
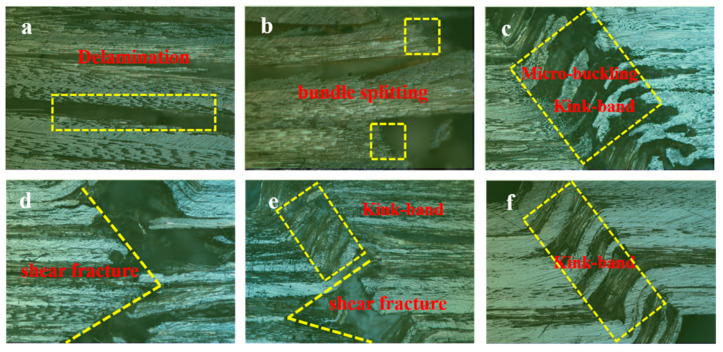
Optical microscope photographs of compression failure sample; (**a**) BF—Desized/PPENK, (**b**) BF-PVP/PPENK, (**c**) BF-PEN/PPENK, (**d**) BF-PPEK/PPENK, (**e**) BF-PPEN/PPENK and (**f**) BF-PPENK/PPENK composites.

As shown in Figure 7a, many interlaminar cracks appeared on the side of the BF—Desized/PPENK, and the cracks propagated between the layers along the direction of BF, which is a typical delamination failure mode, mainly due to the weak interfacial strength [50]. The broken ends of fiber bundles and brittle fracture for the BF-PVP/PPENK composite (Figure 7b) indicated that the bundle splitting was its main failure mode. This is because of the excessively flexible interfacial phase which is easily destroyed under the huge load, and the fiber bundles endure almost all the stress. When the weak links in the fibers are overload and fracture, the cracks rapidly expand to the surrounding fibers and cause the fiber bundles to bend and fracture, frequently accompanied by delamination. The obvious “V”-shaped fracture zone of the BF-PPEK/PPENK composite indicated that its main failure mode was shear fracture (Figure 7d), which is closely related to the strong rigid structures in the PPEK chains; due to this relatively rigid interfacial phase, it prevents the crack from propagating along the direction of fiber and eventually developed an inclined crack. The “Z”-shaped inclined band in the failure morphology of BF-PEN/PPENK was a kink band (Figure 7c), implying the improved interfacial properties, and its formation is mainly caused by the micro-buckling of the fibers. The kink band was also observed on the BF-PPEN/PPENK and BF-PPENK/PPENK composites (Figure 7e,f), but there was another failure mode of shear fracture in the former, which may be attributed to the much better interfacial properties of BF-PPEN/PPENK.

**Figure 8:**
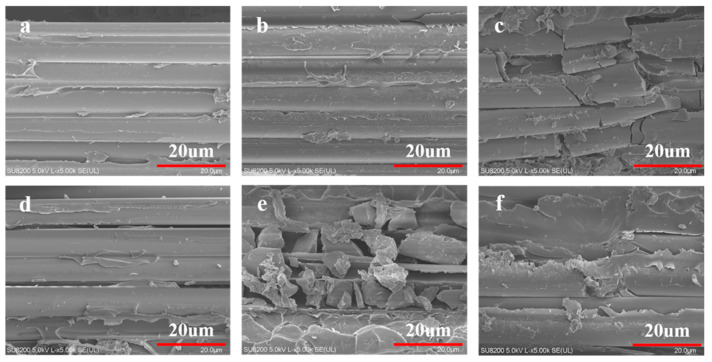
SEM images of compression failure sample; (**a**) BF—Desized/PPENK, (**b**) BF-PVP/PPENK, (**c**) BF-PEN/PPENK, (**d**) BF-PPEK/PPENK, (**e**) BF-PPEN/PPENK and (**f**) BF-PPENK/PPENK composites.

The SEM images indicated that the delamination of the BF—Desized/PPENK composites has a smooth surface, which means that the adhesion between the fibers and matrix is weak (Figure 8a). Compressive failure microtopography of the BF-PVP/PPENK and BF-PPEK/PPENK composites showed no fiber fragments on the delaminated surface and a little plastic deformation (Figure 8b,d), but the compressive strength of the latter was higher than the former. This further confirms that the sizing agent with rigid chain structures has a great effect on the improvement in the compressive properties. Particularly, the abundant BF fragments in Figure 8c,e demonstrated that PEN and PPEN sizing agents were more effective than others in improving the compressive performance of BF/PPENK composites because of the cyano groups and the aromatic rings contained in the molecular chains. Moreover, the compressive strengths of BF-PEN/PPENK and BF-PPEN/PPENK composites were much higher than others, which are attributed to energy dissipation by the fiber crushing. However, even though the BF-PPENK/PPENK compression failure mode was kink band, it did not have many fiber fragments, which can be interpreted as a result of its relatively low compressive properties (Figure 8f).

### 3.8. Flexural Strength and ILSS

**Figure 9:**
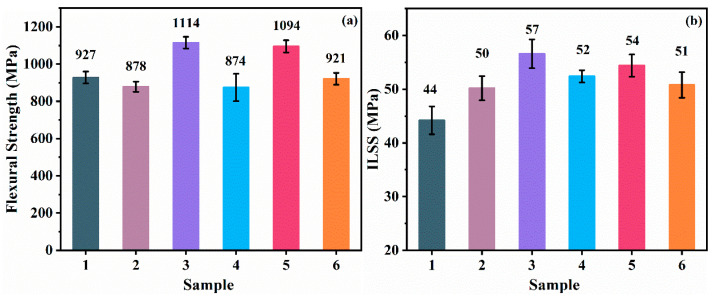
(**a**) Flexural strength and (**b**) interlaminar shear strength of the 1: BF—Desized/PPENK, 2: BF-PVP/PPENK, 3: BF-PEN/PPENK, 4: BF-PPEK/PPENK, 5: BF-PPEN/PPENK and 6: BF-PPENK/PPENK composites.

The flexural strengths of BF-PEN/PPENK and BF-PPEN/PPENK composites were 1114 MPa and 1094 MPa, increased by 20% and 18% compared with BF—Desized/PPENK (927 MPa), respectively (Figure 9a). However, the flexural strengths of BF-PVP/PPENK, BF-PPEK/PPENK and BF-PPENK/PPENK composites were slightly decreased. The interlaminar shear strength (ILSS) can largely reflect the interfacial properties of the composite [53], and the ILSS of BF-PVP/PPENK, BF-PEN/PPENK, BF-PPEK/PPENK, BF-PPEN/PPENK and BF-PPENK/PPENK composites reached 50 MPa, 57 MPa, 52 MPa, 54 MPa, and 51 MPa, which were increased by 13.6%, 29.5%, 18.2%, 22.7%, and 15.9%, respectively (Figure 9b). Therefore, compared with the other four sizing agents, PEN and PPEN are more effective in improving the interfacial properties of the BF/PPENK composites, while the flexible interfacial phase structures constructed by PVP, PPEK and PPENK are not very reasonable. In fact, the flexible interfacial layer has two effects on the mechanical properties of the composite. On the one hand, it can improve the interfacial properties by deformation; on the other hand, the low modulus will reduce the strength. However, when the improvement is not enough to compensate for the weakening, the composite exhibits a decrease in strength. Especially, when the composite is subjected to a bending test, it will be subjected to a variety of forces such as tension, compression and shear, and the effect of the resultant force will make this influence more obvious.

## 4. Conclusions

In this work, five sizing agents (PVP, PEN, PPEK, PPEN and PPENK) were successfully coated on the BF surface to improve the interfacial and mechanical properties of the BF/PPENK composites. The PVP sizing agent can form a flexible interfacial phase with enhanced mechanical properties in BF/PPENK composites, while the BF-PEN/PPENK composite with the PEN sizing agent has the strongest interfacial phase due to the relatively rigid chain structures, and strong polar interaction, *π*-*π* interaction and compatibility of PEN with the PPENK matrix, resulting in the even higher *T_g_* and mechanical properties. Furthermore, for the other three composites with the similar sizing agents containing a phthalazinone structure, BF-PPEN/PPENK exhibited higher tensile strength, tensile modulus and elongation at break than those of BF-PPEK/PPENK and BF-PPENK/PPENK, due to the relatively better film-forming properties and many more polar cyano groups (–CN) of PPEN than those of PPEK and PPENK. In summary, thermoplastic sizing agents with favorable film-forming ability on BF, good compatibility with the matrix, moderate flexibility and strong polar group are preferred for BF-reinforced high-performance thermoplastic composites with improved interfacial and mechanical properties.

## Figures and Tables

**Table 1:** Solubility parameter values of the PPENK matrix resin and sizing agents ^a^.

Sample	***δ**_**d**_*	***δ**_**p**_*	***δ**_**h**_*	***δ**_**t**_*	|*δ_t_* − *δ_t,matrix_*|	|*δ_p_* − *δ_p,matrix_*|
PPEK	19.51	5.02	6.83	21.27	0.26	0.8
PPENK	19.44	5.82	7.21	21.53	0	0
PPEN	19.35	6.77	7.65	21.88	0.35	0.95
PVP	16.98	11.35	8.46	22.11	0.58	5.53
PEN	19.82	7.16	5.62	21.81	0.28	1.34

^a^ where *δ_t_*, *δ_d_*, *δ_p_* and *δ_h_* represent the total solubility parameter, dispersion component, polarization component and hydrogen bond component, respectively.

## Data Availability

Not applicable.

## Supplementary Materials

The following are available online at https://www.mdpi.com/article/10.3390/polym14142947/s1. Figure S1 TGA curves of the sizing agents of PVP, PEN, PPEK, PPEN and PPENK. Figure S2 Synthesis of LMW PEN. Figure S3 The preparation process flow diagram of composite laminates. Figure S4 XPS spectra of the desized and re-sized basalt fibers; (a) survey scan spectra and (b) narrow scan spectra in the N 1s regions. Figure S5 Loss modulus and tan δin curves of BF-Desized/PPENK, BF-PVP/PPENK, BF-PEN/PPENK, BF-PPEK/PPENK, BF-PPEN/PPENK and BF-PPENK/PPENK composites. Figure S6 Images of overall morphology of tensile failure; (a) BF-Desized/PPENK, (b) BF-PVP/PPENK, (c) BF-PEN/PPENK, (d) BF-PPEK/PPENK, (e) BF-PPEN/PPENK and (f) BF-PPENK/PPENK composites. Figure S7 Flexural modulus of the 1: BF-Desized/PPENK, 2: BF-PVP/PPENK, 3: BF-PEN/PPENK, 4: BF-PPEK/PPENK, 5: BF-PPEN/PPENK and 6: BF-PPENK/PPENK composites. Table S1 Tensile and compression tests of BF/PPENK composites. Table S2 Quantification of the atomic chemical composition of the BFs. Table S3 DMA test results of BF/PPENK composites. Table S4 Tensile and compression tests of BF/PPENK composites. Table S5 Flexural and ILSS tests of BF/PPENK composites.
