# Supplementary material for: Highly Enhancing the Interfacial and Mechanical Properties of Basalt Fiber/Poly(phthalazinone ether nitrile ketone) Composite by Thermoplastic Sizing Agents with Different Structures
Source: Polymers (Basel). 2022 Jul 21;14(14):2947. doi: 10.3390/polym14142947 (PMC9315541; doi:10.3390/polym14142947)
Supplement: Supplementary file 1 [file polymers-14-02947-s001.zip › polymers-1781770-supplementary.pdf]

**Supplementary Information for**

# Highly enhanced the interfacial and mechanical properties of basalt fiber/poly(phthalazinone ether nitrile ketone) composite by thermoplastic sizing agents with different structures

Hang Jia[a,b], Cheng Liu[a,b,d *], Yue Qiao[a,b], Yu Zhang[a,b], Kaiyuan Fan[b], Manxia Zhang[c], Xigao Jian[a,b,d]

[a] State Key Laboratory of Fine Chemicals, Dalian University of Technology, Dalian, 116024, People's Republic of China;

[b] Department of Polymer Science & Materials, Dalian University of Technology, Dalian, 116024, People's Republic of China;

[c] Department of Environmental Science & Engineering, Dalian Maritime University, Dalian, 116026, People's Republic of China;

[d] Dalian Basalt Fiber Resin Matrix Composite Engineering Research Center, Dalian, 116012, People's Republic of China.

[*] Corresponding author. Tel.: +86-411-84986191.

Email address: liuch1115@dlut.edu.cn (C. Liu)

**Supplementary Figures:**

Figure S1. TGA curves of the sizing agents of PVP, PEN, PPEK, PPEN and PPENK.

Figure S2. Synthesis of LMW PEN.

Figure S3. The preparation process flow diagram of composite laminates.

Figure S4 XPS spectra of the desized and re-sized basalt fibers; (a) survey scan spectra and (b) narrow scan spectra in the N 1s regions.

Figure S5. Loss modulus and $tan\ \delta_{in}$ curves of BF-Desized/PPENK, BF-PVP/PPENK, BF-PEN/PPENK, BF-PPEK/PPENK, BF-PPEN/PPENK and BF-PPENK/PPENK composites.

Figure S6. Images of overall morphology of tensile failure; (a) BF-Desized/PPENK, (b) BF-PVP/PPENK, (c) BF-PEN/PPENK, (d) BF-PPEK/PPENK, (e) BF-PPEN/PPENK and (f) BF-PPENK/PPENK composites.

Figure S7. Flexural modulus of the 1: BF-Desized/PPENK, 2: BF-PVP/PPENK, 3: BF-PEN/PPENK, 4: BF-PPEK/PPENK, 5: BF-PPEN/PPENK and 6: BF-PPENK/PPENK composites.

Table S1. Tensile and compression tests of BF/PPENK composites.

Table S2 Quantification of the atomic chemical composition of the BFs.

Table S3 DMA test results of BF/PPENK composites

Table S4. Tensile and compression tests of BF/PPENK composites

Table S5. Flexural and ILSS tests of BF/PPENK composites.

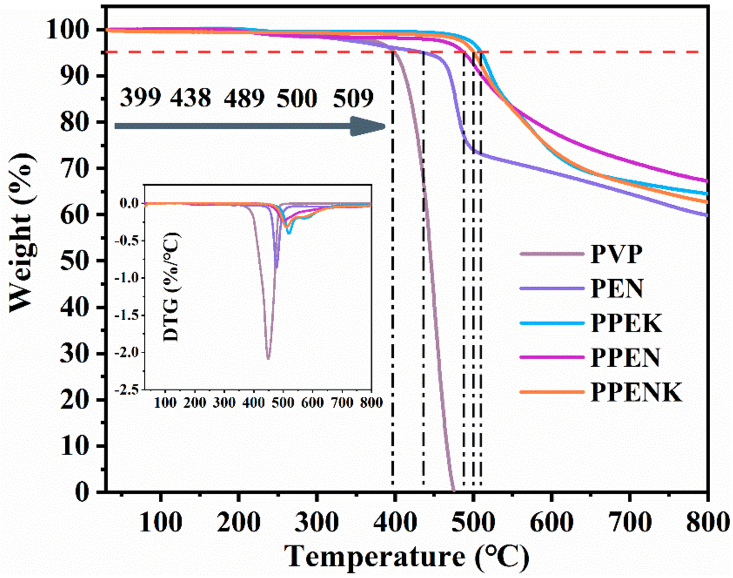


Figure S1. TGA curves of the sizing agents of PVP, PEN, PPEK, PPEN and PPENK.

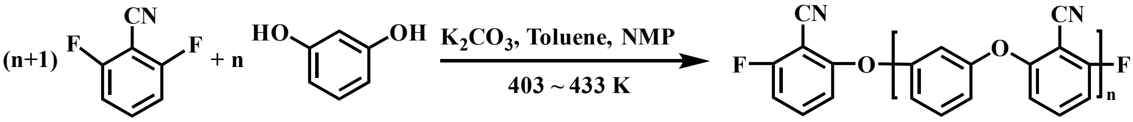


Figure S2. Synthesis of LMW PEN.

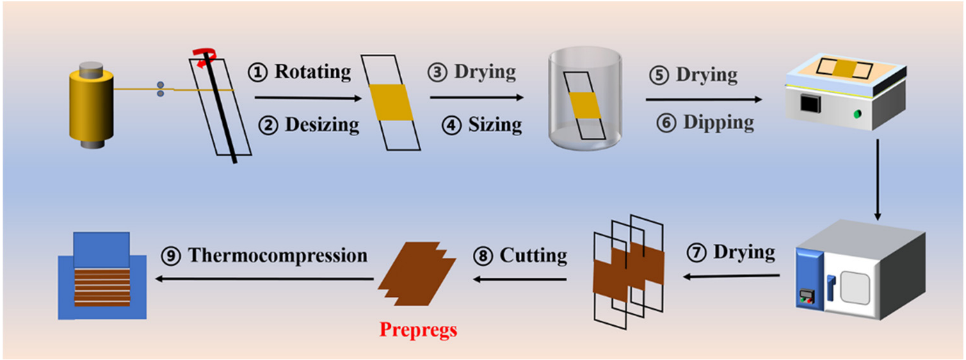


Figure S3. The preparation process flow diagram of composite laminates.

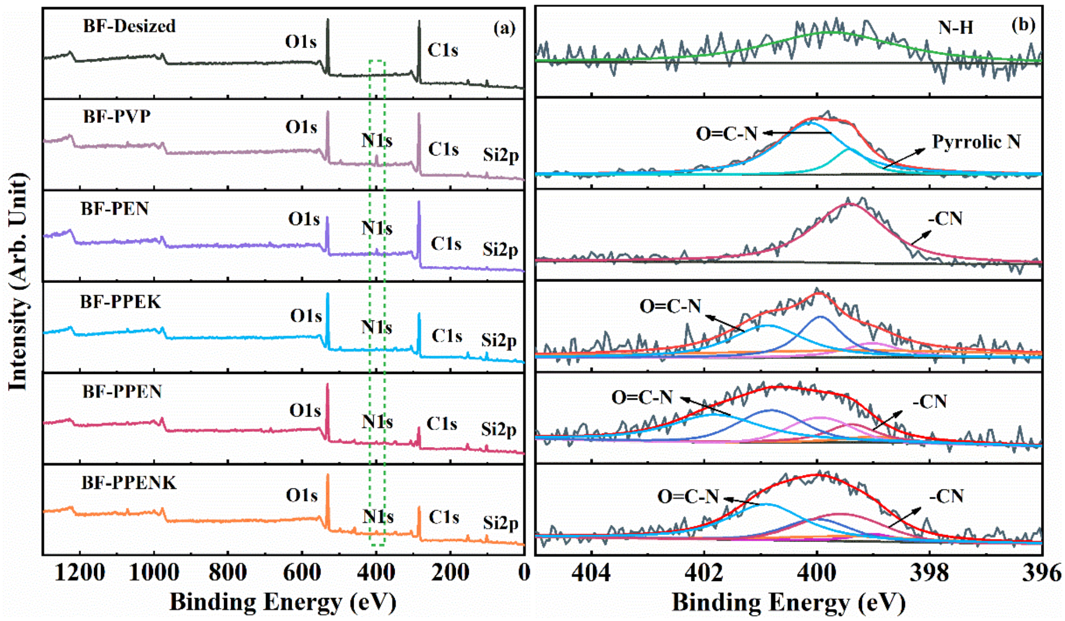


Figure S4. XPS spectra of the desized and re-sized basalt fibers; (a) survey scan spectra and (b) narrow scan spectra in the N 1s regions.

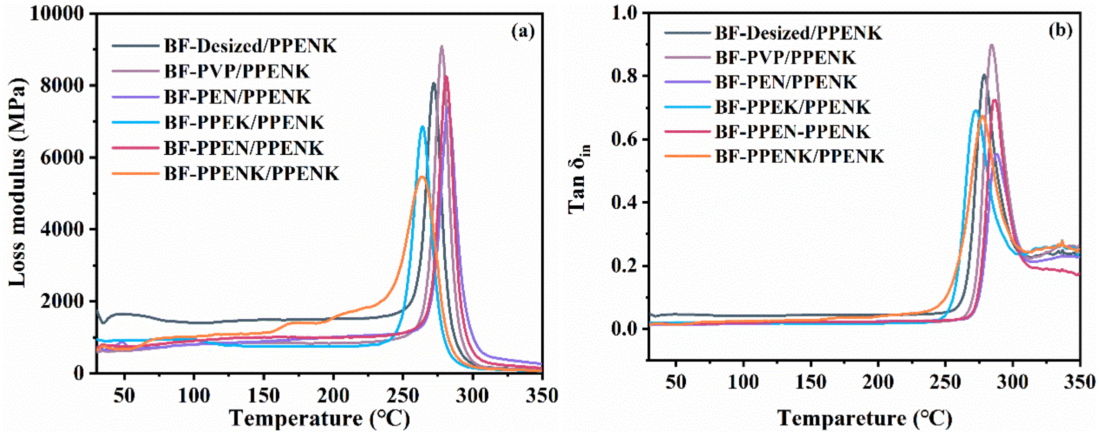


Figure S5. Loss modulus and tan$\delta_{in}$ curves of BF-Desized/PPENK, BF-PVP/PPENK, BF-PEN/PPENK, BF-PPEK/PPENK, BF-PPEN/PPENK and BF-PPENK/PPENK composites.

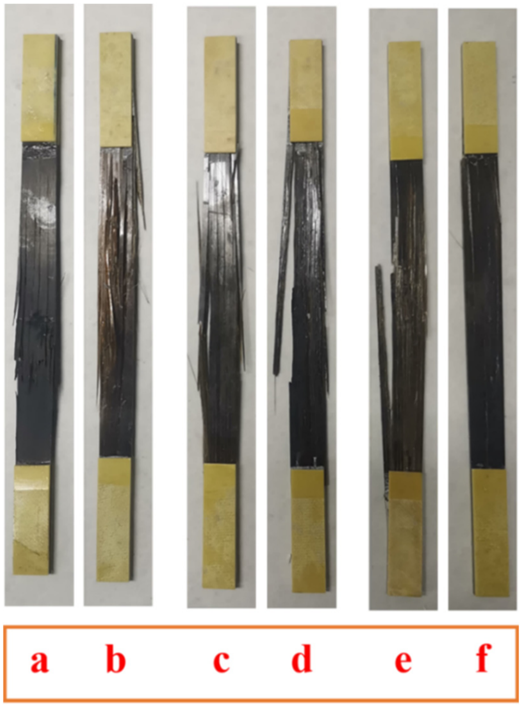


Figure S6. Images of overall morphology of tensile failure; (a) BF-Desized/PPENK, (b) BF-PVP/PPENK, (c) BF-PEN/PPENK, (d) BF-PPEK/PPENK, (e) BF-PPEN/PPENK and (f) BF-PPENK/PPENK composites.

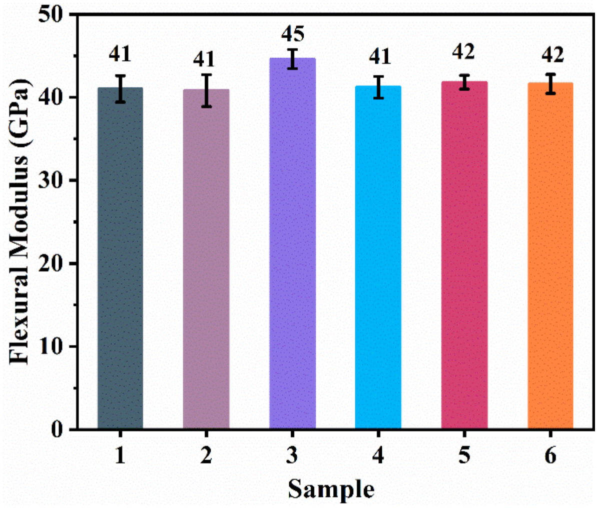


Figure S7. Flexural modulus of the 1: BF-Desized/PPENK, 2: BF-PVP/PPENK, 3: BF-PEN/PPENK, 4: BF-PPEK/PPENK, 5: BF-PPEN/PPENK and 6: BF-PPENK/PPENK composites.

Table S1. Tensile and compression tests of BF/PPENK composites

| Radical | $F_{di}$ | $F_{pi}$ | $E_{hi}$ | $V_i$ |
|---|---|---|---|---|
| | $J^{1/2}\ cm^{3/2}\ mol^{-1}$ | $J^{1/2}\ cm^{3/2}\ mol^{-1}$ | $J\ mol^{-1}$ | $cm^3\ mol^{-1}$ |
| =N- | 347 | 712 | 2816 | 5.0 |
| $-C_6H_4-$ | 1270 | 110 | 0 | 65.5 |
| -CN | 430 | 1100 | 2500 | 23.1 |
| -O- | 100 | 400 | 3000 | 10 |
| -CO- | 290 | 770 | 2000 | 13.4 |
| -N- | 20 | 800 | 5000 | 6.7 |
| =C- | 70 | 0 | 0 | 7.18 |
| =CH- | 200 | 0 | 0 | 13.18 |
| $-CH_2-$ | 270 | 0 | 0 | 15.55 |
| -CH- | 80 | 0 | 0 | 9.56 |
| Ring | 190 | - | - | - |

Table S2 Quantification of the atomic chemical composition of the BFs.

| Fiber | Chemical composition (%) | | | | | | |
|---|---|---|---|---|---|---|---|
| | C | O | N | Al | C/Al | O/Al | N/Al |
| BF-Ddesized | 65.28 | 25.58 | 0.99 | 1.88 | 34.72 | 13.61 | 0.53 |
| BF-PVP | 45.91 | 42.8 | 7.11 | 0.64 | 71.73 | 66.88 | 11.11 |
| BF-PEN | 53.68 | 38.56 | 5.27 | 0.48 | 111.83 | 80.33 | 10.98 |
| BF-PPEK | 35.83 | 56.02 | 1.71 | 1.07 | 33.83 | 52.35 | 1.60 |
| BF-PPEN | 21.25 | 67.96 | 2.47 | 1.34 | 15.85 | 50.72 | 1.84 |
| BF-PPENK | 28.16 | 61.39 | 3.22 | 1.28 | 22.00 | 47.96 | 2.52 |

Table S3 DMA test results of BF/PPENK composites

| Composites | Storage modulus (30 °C, GPa) | $T_g$ (°C) | tan$\delta_{max}$ |
|---|---|---|---|
| BF-Desized/PPENK | 36.65 | 279 | 0.8251 |
| BF-PVP/PPENK | 46.78 | 285 | 0.9046 |
| BF-PEN/PPENK | 50.14 | 288 | 0.5559 |
| BF-PPEK/PPENK | 45.74 | 273 | 0.7489 |
| BF-PPEN/PPENK | 49.50 | 286 | 0.7255 |
| BF-PPENK/PPENK | 44.81 | 277 | 0.7189 |

Table S4. Tensile and compression tests of BF/PPENK composites

| Composites | Tensile test | | | Compression test | | |
|---|---|---|---|---|---|---|
| | Strength MPa | Modulus GPa | Strain % | Strength MPa | Modulus GPa | Breaking train % |
| BF-Desized/PPENK | 547±23 | 28±1.4 | 2.25±0.23 | 403±52 | 34±3.1 | 1.61±0.21 |
| BF-PVP/PPENK | 751±22 | 34±0.8 | 2.78±0.09 | 490±25 | 40±3.1 | 1.44±0.13 |
| BF-PEN/PPENK | 778±65 | 33±0.8 | 2.86±0.24 | 600±41 | 36±1.5 | 2.24±0.15 |
| BF-PPEK/PPENK | 662±61 | 28±1.7 | 2.57±0.27 | 568±39 | 40±0.5 | 1.81±0.24 |
| BF-PPEN/PPENK | 722±39 | 31±0.6 | 2.76±0.22 | 695±34 | 43±0.8 | 2.03±0.15 |
| BF-PPENK/PPENK | 608±58 | 29±1.8 | 2.71±0.14 | 452±57 | 36±2.2 | 1.84±0.28 |

Table S5. Flexural and ILSS tests of BF/PPENK composites

| Composites | Flexural test | | ILSS test |
| --- | --- | --- | --- |
| | Strength (MPa) | Modulus (GPa) | Strength (MPa) |
| BF-Desized/PPENK | 927±32 | 41±1.6 | 44±2.6 |
| BF-PVP/PPENK | 878±26 | 41±1.9 | 50±2.3 |
| BF-PEN/PPENK | 1115±32 | 45±1.1 | 57±2.7 |
| BF-PPEK/PPENK | 874±73 | 41±1.3 | 52±1.1 |
| BF-PPEN/PPENK | 1095±32 | 42±0.8 | 54±2.1 |
| BF-PPENK/PPENK | 921±32 | 42±1.1 | 51±2.4 |
